# Naked Mole-Rats Demonstrate Profound Tolerance to Low Oxygen, High Carbon Dioxide, and Chemical Pain

**DOI:** 10.3390/ani13050819

**Published:** 2023-02-24

**Authors:** Vince G. Amoroso, Aishi Zhao, Isabel Vargas, Thomas J. Park

**Affiliations:** Department of Biological Sciences, University of Illinois at Chicago, Chicago, IL 60607, USA

**Keywords:** naked mole-rat, hypoxia, anoxia, pain, Na_V_1.7, HIF-1a, VEGF, Substance P, KCC2

## Abstract

**Simple Summary:**

Naked mole-rats live in crowded underground burrows where concentrations of oxygen can be low and concentrations of carbon dioxide can be high. Accordingly, this species is tolerant of low oxygen levels and high carbon dioxide levels, which would be deadly to most surface dwellers. The current article reviews what we know about these unusual tolerances and their underlying mechanisms. Understanding these mechanisms could lead to new strategies for treating human disorders related to low oxygen and high carbon dioxide, as experienced, for example, during a heart attack.

**Abstract:**

Naked mole-rats (*Heterocephalus glaber*) are very unusual among subterranean mammals in that they live in large colonies and are extremely social, spending large amounts of time gathered together in underground nests more than a meter below the surface. Many respiring individuals resting in deep, poorly ventilated nests deplete the oxygen supply and increase the concentration of carbon dioxide. Consistent with living in that atmosphere, naked mole-rats tolerate levels of low oxygen and high carbon dioxide that are deadly to most surface-dwelling mammals. Naked mole-rats appear to have evolved a number of remarkable adaptations to be able to thrive in this harsh atmosphere. In order to successfully survive low oxygen atmospheres, they conserve energy utilization by reducing the physiological activity of all organs, manifest by reduced heart rate and brain activity. Amazingly, they resort to the anaerobic metabolism of fructose rather than glucose as a fuel to generate energy when challenged by anoxia. Similarly, high carbon dioxide atmospheres normally cause tissue acidosis, while naked mole-rats have a genetic mutation preventing both acid-induced pain and pulmonary edema. Together, these putative adaptations and the tolerances they provide make the naked mole-rat an important model for studying a host of biomedical challenges.

## 1. Introduction

Naked mole-rats are small rodents endemic to the arid and semi-arid regions of the sub-Saharan northeastern horn of Africa. Here, they live in a complex maze of underground burrows reaching more than 3000 m in length [1] and reaching depths of more than 1.8 m [2]. They primarily forage and disperse through excavating tunnels, an energetically costly activity, exacerbated by food being sparse and patchily distributed [2]. Naked mole-rats meet all their energy and water requirements through the foods they consume and the metabolic water they generate [1]. In addition to having to contend with the daunting problems associated with foraging below ground, they may also encounter numerous physiological challenges imposed by underground atmospheric conditions. Both gas and heat exchange are dependent on the diffusion and porosity properties of the soil and may be impaired. As fossil evidence suggests they have occupied this subterranean niche since the early Miocene [3,4], it is not surprising that they have evolved a fascinating montage of biological traits well suited to the harsh conditions they encounter in their hot, humid, and poorly ventilated sealed maze of burrows (for a review, see [5]). These include having a lower body temperature (32 ℃) than most mammals and a reduced basal metabolic rate for their body size. Not surprisingly, when they are housed outside of the warm and humid confines of these equatorial burrows, and lacking an insulatory pelage, naked mole-rats are poor thermoregulators, unable to counter the high rates of heat loss with effective endothermic mechanisms and rather rely on communal huddling to keep warm [5,6].

Naked mole-rats live in large groups of up to 295 individuals [1]. Reproduction is confined to a single breeding female and 1–3 breeding males within the colony, and there is a high reproductive skew such that less than 1% of all individuals within the colony get the opportunity to breed over their lifetime, contributing to their description as one of only two eusocial mammals [7,8]. Living in large colonies, they have distinct, culturally transmitted vocal dialects [9], despite having a non-functional cochlear amplifier and associated poor auditory thresholds [10]. Many of their other adaptive traits to this environmental niche may also protect against numerous disease states, including cancer, neurodegeneration, and cardiovascular disease, contributing to prolonged healthy aging and extreme longevity [11]. Naked mole-rats are extremely long-lived for their body size, living approximately six times longer than predicted allometrically. Intriguingly, unlike every other mammalian species studied to date, in which after the age of sexual maturity there is an exponential increase in risk of dying, naked mole-rats do not. Rather, death is random, with similar likelihoods of dying at 2 and 20 years of age [11]. In other words, they do not show an increased risk of dying with advancing age, defying Gompertzian fundamental laws of mortality. This stochastic pattern of age-independent risk of dying is accompanied by a negligible senescence phenotype, maintaining physiological function well into their fourth decade [12], as well as retaining many youthful paedomorphic features, including high oxygen affinity fetal hemoglobin in adult life [13]. These and many other unusual qualities make the naked mole-rat an interesting model species with an abundance of recent genomic and epigenetic aging clock data available, facilitating the creation of explanatory models [14,15,16,17]. 

In this review, we will cover significant adaptations that the naked mole-rat has evolved, likely due to living in their unique subterranean niche [2]. Their communal lifestyle, while facilitating better foraging success, is not without problems. The animals rest and respire together in deep nests where diffusion through the soil is poor, creating an atmosphere that is both hypoxic and hypercapnic. Naked mole-rats are very tolerant of these environmental challenges [2,18,19], and here we report on some of the underlying mechanisms that have been identified to date [19,20,21,22,23].

## 2. Tolerance to Oxygen Deprivation

**Figure 1:**
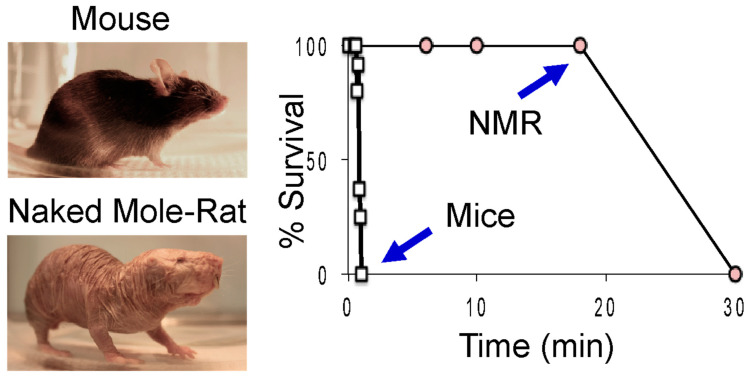
Naked mole-rats survive much longer than mice in 0% oxygen. The graph shows the percent of animals able to survive a given duration of anoxia. Different animals were tested for each time point. NMR = naked mole-rat. These data are from [19].

Naked mole-rats demonstrate a profound tolerance to oxygen deprivation when compared to mice [19]. This includes resilience against exposure to low oxygen (hypoxia) atmospheres as well as no oxygen (anoxia). While all mice can survive a 30 s exposure to anoxia, none survive a 1 min exposure (Figure 1). In contrast, all naked mole-rats survived an 18 min exposure to anoxia, although no naked mole-rats survived a 30 min exposure.

**Figure 2:**
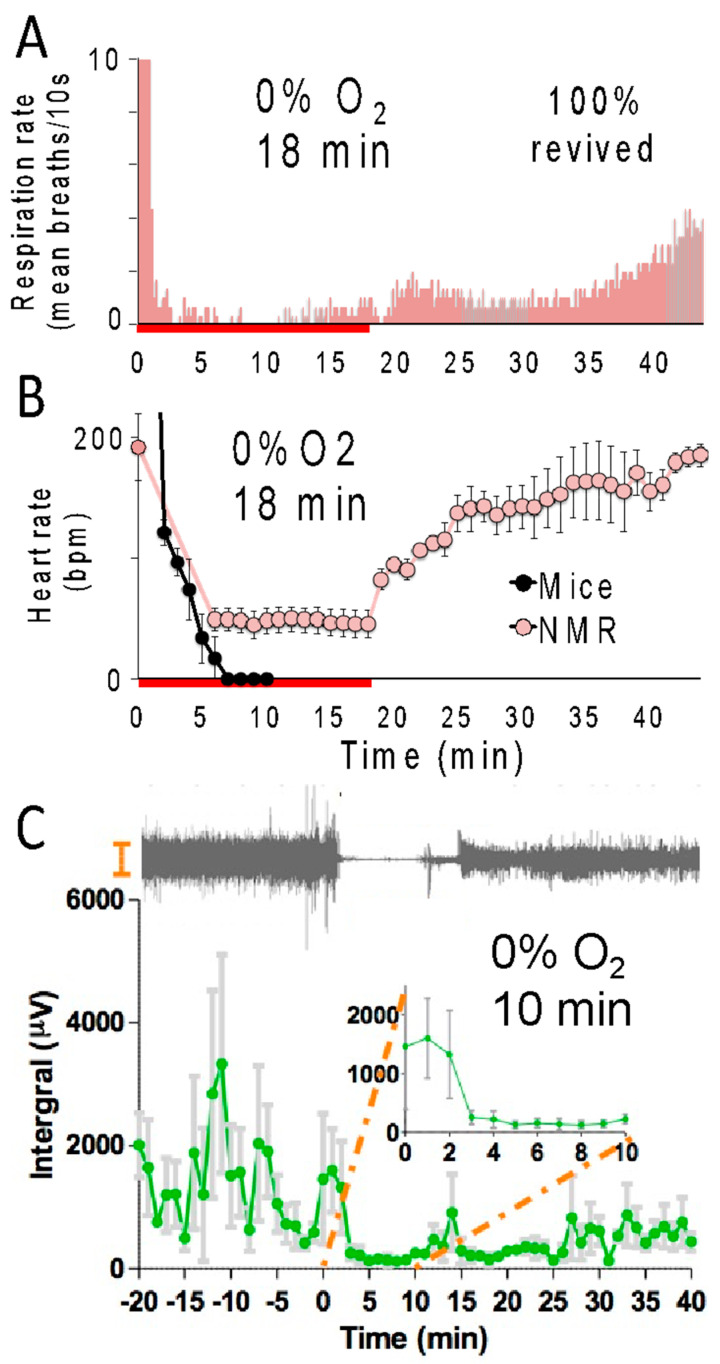
Naked mole-rats show reduced physiological activity during anoxia. Average respiration rate (**A**) and heart rate (**B**) of naked mole-rats during an 18 min anoxia exposure (red line below the X-axis) followed by normoxia. These data are from [19]. (**C**) Top, an example electroencephalography (EEG) trace from a naked mole-rat before, during, and after a 10 min of exposure to anoxia. Bottom: average brain activity for naked mole-rats before, during, and after a 10 min exposure to anoxia. This data is from [25]. N = 3 for panels (**A**–**C**).

During anoxia, naked mole-rats drastically reduced physiological activities, presumably to conserve energy expenditure [19,24,25,26]. Within 30 s of exposure to anoxia, animals lose consciousness, and their respiration rate is drastically depressed (Figure 2A), suggestive of a lower metabolic rate [13,18]. The red line below the X-axis indicates the time course of anoxia exposure. After exposure to anoxia and a return to normoxic room air, the respiration rate increased toward baseline levels. During anoxic exposure, the heart rate of naked mole-rats decreased from about 200 beats per minute to about 50 beats per minute and then recovered to baseline levels of activity under normoxic recovery conditions (Figure 2B). This is consistent with data collected by Ilacqua et al. [24], showing that under acute hypoxia and anoxia, naked mole-rats reduce metabolic rates by decreasing locomotion and body temperature. For mice, the electrical activity in mouse hearts was not detectable in the electrocardiogram after 6 min of exposure to anoxia. Another physiological metric is brain activity as measured by electroencephalography (EEG) of the caudate putamen. It was found that brain activity in naked mole-rats was dramatically reduced during a 10 min exposure to anoxia [25] (Figure 2C). The EEG revealed that within 3–5 min, brain activity had declined and remained at these low levels for the duration of anoxic exposure. EEG recordings were acquired using a four-channel Pinnacle Technology EEG system, and electrodes were positioned stereotaxically. 

It is important to note that naked mole-rats in the wild are likely to encounter hypoxia, not full anoxia. Park et al. [19] showed that naked mole-rats tolerate 5% O_2_ for at least 5 h, whereas mice cannot survive for more than about 12 min. Additionally, Ilacqua et al. [24] tested naked mole-rats with 9%, 7%, 5%, and 3% O_2_ and showed an incremental decline in activity with increasing severity of hypoxia. 

**Figure 3:**
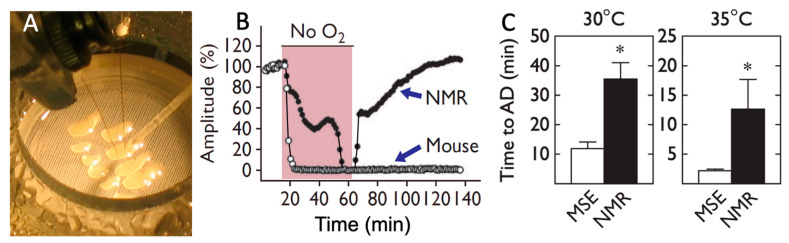
Hippocampal brain slices from naked mole-rats maintain functionality during anoxia much longer than slices from mice. (**A**) This photograph shows the interface recording chamber where slices were tested (photo by Thomas Park). (**B**) The graph shows evoked potential amplitude before, during, and after exposure to anoxia for one slice from a mouse and one slice from a naked mole-rat. The shaded region corresponds to when anoxia was applied; at other times, the brain slices were exposed to normoxia. (**C**) Mean time to anoxic depolarization for slices from mice (MSE), n = 19, and naked mole-rats (NMR), n = 13. * *p* < 0.01. These data are from [27].

Additional studies using electrophysiological assessments of hippocampal brain slices in an interface recording chamber reported intrinsic brain tolerance to anoxia in naked mole-rats [27,28], see Figure 3A. Evoked potential data for one slice from a mouse and one slice from a naked mole-rat are illustrated in Figure 3B, wherein the slices were stimulated alternately every 10 s. After reaching a stable baseline, the slices were exposed to anoxia until both slices lost function (shaded area). In these examples, the slice from a naked mole-rat took significantly longer to experience anoxic depolarization (AD) and loss of detectable functionality. After reoxygenation, naked mole-rat slices showed recovery of synaptic transmission, while mouse slices did not. Given that naked mole-rats and mice have disparate body temperatures (32 °C and 37 °C, respectively), studies were undertaken at waterbath temperatures approximating their divergent body temperatures (Figure 3C). While waterbath temperature greatly affects the time to loss of synaptic function and transmission, it should be noted that at both temperatures, the naked mole-rats showed a significantly longer time to loss of detectable functionality. 

**Figure 4:**
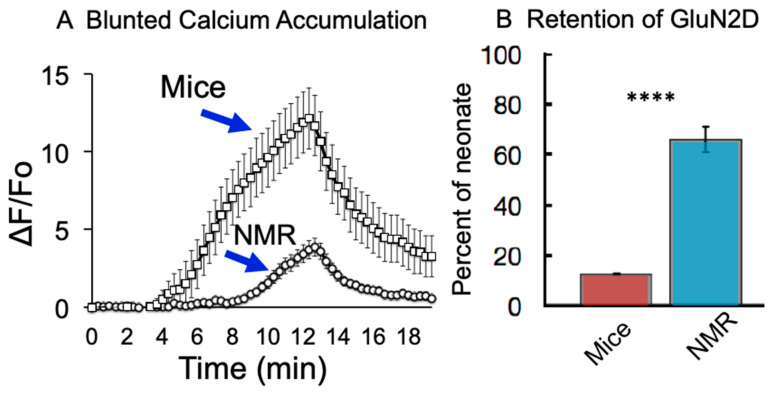
Reduced calcium accumulation from hypoxia in naked mole-rat hippocampal cells (**A**) and greater retention of GluN2D in naked mole-rats compared to mice (**B**) **** *p* < 0.0001. (**A**) is from [29], and (**B**) is from [35].

Naked mole-rat brain cells show a much lower accumulation of intracellular calcium during hypoxia compared to cells from mice [29]. One detrimental effect of hypoxia is that it triggers an increase in intracellular calcium concentration, resulting in the activation of several toxic downstream signaling cascades [30,31]. Unlike the brain slices from mice that show a significant increase in calcium influx during hypoxic challenge, that observed in mole-rat brain slices is considerably attenuated (Figure 4A).

One of the major avenues for neuronal calcium entry is the NMDA receptor (NMDAR). The NMDA receptor is a fundamental building block of neuronal processing, being a central mediator of Hebbian learning and integral to the induction of Long-term Potentiation (LTP) and Long-term Depression (LTD) of neurons. Once the NMDAR is opened, it is notable for its large calcium current that activates second messenger pathways within the neuron. Although calcium entry is necessary for many forms of synaptic plasticity and physiological behavior of the NMDAR, it is highly regulated in the cell, and errant entry during times of overactivation can quickly lead to excitotoxicity [32]. The canonical NMDAR is composed of several subunits, with each receptor being a tetramer that is composed of two requisite NR1 (NMDA receptor, subunit 1) subunits and two subunits of a combination of GluN2A, GluN2B, GluN2C, or GluN2D. Bickler et al. found that the GluN2D subunit reduces its mean open time during hypoxia, reducing hypoxia-induced calcium accumulation [33]. It was demonstrated that calcium accumulation and NMDAR currents were reduced under hypoxia in NMDARs composed of the GluN2D subunit. This alteration in the opening of these channels likely provides a mechanistic contribution to the greater tolerance of hypoxic conditions.

NMDAR subunit composition is age- and region-dependent in most mammals, but globally the proportion of GluN2B and GluN2D decreases with maturity [34]. Adult mice retain only about 10% of the hypoxia-resistant GluN2D subunit compared to neonate mice (Figure 4B). On the other hand, adult naked mole-rats retain about 66% of these receptors with GluN2D subunits that close under hypoxia compared to neonate naked mole-rats [35]. It is believed that this neotenous feature of the naked mole-rat is an adaptation to the hypoxic environment described here, where evolution has favored the trade-off of kinetic characteristics idiosyncratic to the GluN2D subunit, such as lower conductance for a diminished entry of calcium, as more optimal at their point on the fitness landscape.

**Figure 5:**
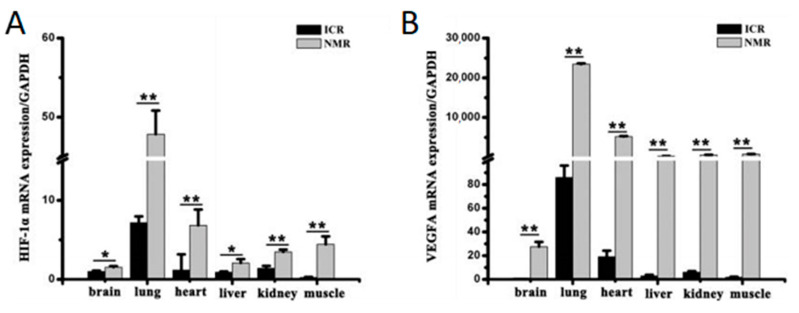
Quantified levels of HIF-1a mRNA in naked mole-rats compared to ICR mice are shown in (**A**), where a significant increase in expression was seen in all six measured tissues. Significant increases were also seen in VEGFA mRNA expression in the naked mole-rat across the same tissues as shown in (**B**). * *p* < 0.05, ** *p* < 0.01. These data are from [36].

Unique molecular adaptations to hypoxia have also been discovered in the naked mole-rat. These include findings by Xiao et al. that Hypoxia Inducible Factor 1 alpha (HIF-1a) and Vascular Endothelial Growth Factor A (VEGFA) are significantly upregulated in naked mole-rat tissues compared to ICR (Institute of Cancer Research) control mice (Figure 5A,B) [36]. Since the discovery in 1995 of HIF-1a by Semenza [37] and its functional role as a molecular sensor of oxygen tension, HIF-1a has played a central mechanistic role in hypoxia research. HIF is hydroxylated in an oxygen-dependent reaction on conserved prolines, allowing for its recognition by Von Hippel-Lindau (VHL), a substrate binder component of the E3 ubiquitin ligase, and thereby targeted for the rapid proteasomal destruction of HIF under normoxic conditions [38]. Under hypoxic conditions, this hydroxylation cannot occur, and HIF levels remain stable and translocate into the nucleus. This oxygen-dependent transcriptional activator regulates the expression of over 100 genes and thereby provides a central hub for deploying the appropriate hypoxic response in the various organ systems [39]. In this manner, HIF regulates biochemical responses to oxygen deprivation by modulating the expression of genes involved in glucose uptake, energy metabolism, as well as cell proliferation and cell death [40]. Xiao et al. have found that even under the normoxic conditions found in the laboratory, naked mole-rat expression of HIF-1a is significantly elevated in several tissues, with concomitant increases in VEGFA, which controls angiogenesis and oxygen delivery [36] (Figure 5B). These responses are undoubtedly of benefit to the naked mole-rat, which experiences bouts of hypoxia in their crowded nest environments and perhaps other burrow segments in the wild.

Genetic analysis of the naked mole-rat genome by Kim et al. uncovered two intriguing mutations hardwired into the HIF-VHL system [16]. Within the HIF-1 sequence at location 407, the naked mole-rat has exchanged threonine for isoleucine (T407I). This point mutation was proposed to be significant as, within the 3D structure of HIF-1, it lies within the VHL-binding domain [16], altering substrate binding for ubiquitination. In addition, it was found that naked mole-rat VHL contains a mutation at residue 166 (V166I). This site is functionally important since other mutations found here are correlated with a pathological outcome, Von Hippel-Lindau disease, due to a weakened interaction in the HIF-VHL complex resulting in reduced HIF1a degradation, a longer HIF-1 half-life, and concomitantly higher constitutive levels [41,42]. These mutations may contribute significantly to the higher constitutive levels observed by Xiao et al. [36] and are consistent with an evolutionary adaptation to tolerate hypoxic conditions as described in this review.

**Figure 6:**
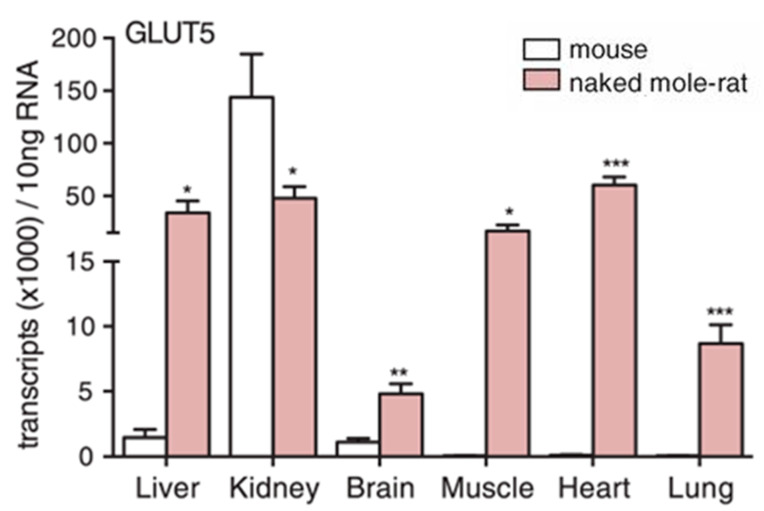
Naked mole-rats have significantly higher levels of the fructose transporter, Glut5, in most organs compared to mice. * *p* < 0.05, ** *p* < 0.01, *** *p* < 0.001. This data is from [19].

Naked mole-rats have another unusual systemic adaptation for dealing with hypoxia and anoxia that involves undergoing drastic metabolic rewiring, which enables the anaerobic glycolysis of fructose [19,43]. In this study, it was demonstrated that naked mole-rats express GLUT5 (solute carrier family 2 member 5), the fructose transporter, at significantly higher levels (>10-fold) in their brain and heart cells compared to mice (Figure 6). Naked mole-rats also showed a substantial and significant increase in fructose in the blood during anoxia, whereas mice did not. Levels of ketohexokinase (KHK), which phosphorylates fructose into fructose-1-phosphate (F1P), were significantly elevated in the naked mole-rat. Additionally, in brain slices exposed to hypoxia, a metabolic flux analysis demonstrated that fructose-derived carbons accumulated at a significantly higher level within many glycolytic intermediates in slices from naked mole-rats compared to slices from mice, indicating the metabolism and incorporation of fructose into naked mole-rat metabolism. Finally, in a demonstration of the physiological utility of this metabolic rewiring, brain slices and isolated hearts from naked mole-rats and mice were tested by switching glucose to fructose in the bath, and their functionality was measured. In both the brain and heart preparations, naked mole-rats retained significantly better performance with fructose compared to mice.

A model was proposed in which glucose metabolism is blocked under near-anoxia by inhibition at the rate-limiting step of phosphofructokinase (PFK). This feedback inhibition is induced by protons, ATP binding, or downstream intermediates such as citrate [19]. The authors put forward that the naked mole-rat has evolved to efficiently interface with and make use of fructose under near-anoxic conditions to bypass the metabolic block at PFK that glucose suffers from and provide for a continued, albeit less efficient, energetic source under these trying periods.

**Figure 7:**
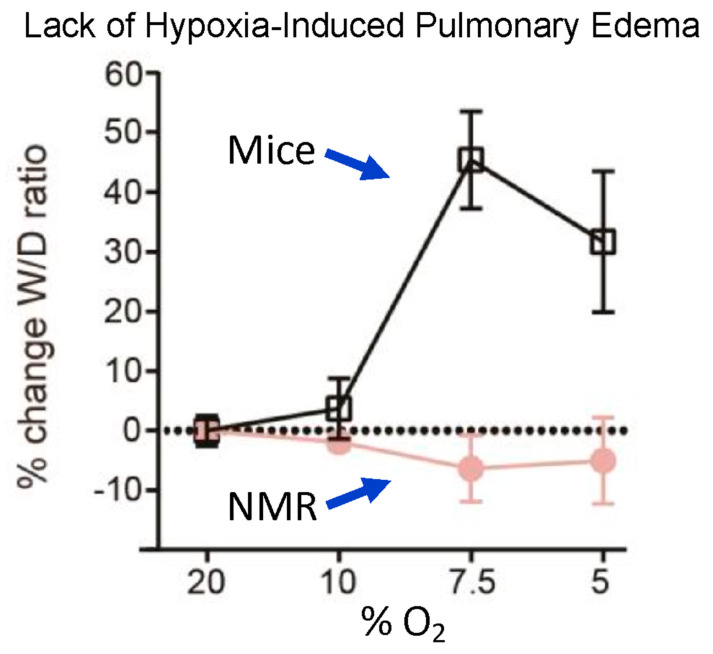
Lack of hypoxia-induced pulmonary edema in naked mole-rats. The curves show the percent change in lung wet—to—dry ratio as a function of the percent oxygen in the atmosphere. Animals were exposed to a given atmosphere for 15 min and then anesthetized with isoflurane and euthanized by rapid decapitation prior to removing and weighing the lungs. This figure is from [19].

Another detrimental effect of hypoxia in most mammals is a rapid buildup of fluid in the lungs known as pulmonary edema. This is caused by altered sympathetic tone, vasoconstriction, and pulmonary hypertension, as well as altered alveolar endothelial and epithelial permeability to water and macromolecules, causing an increased permeability across the alveolar barrier and the capillaries to leak into the alveolar sacs and interstitial spaces that overwhelm the alveolar reabsorption capacity [44]. This is manifested by a change in the wet/dry weight of excised lung tissue. Naked mole-rats appear to be completely immune to this effect. Park et al. [19] exposed naked mole-rats and mice to normoxia (20% O_2_) or hypoxia (10%, 7.5%, or 5% O_2_) for 15 min. Mice showed severe pulmonary edema for hypoxic atmospheres of 7.5% and 5% O_2_, while naked mole-rats showed no edema at either of these low O_2_ concentrations. (Figure 7). Currently, the mechanisms facilitating the lack of hypoxia-induced edema in naked mole-rats are unknown.

## 3. Tolerance to High Concentrations of CO_2_

**Figure 8:**
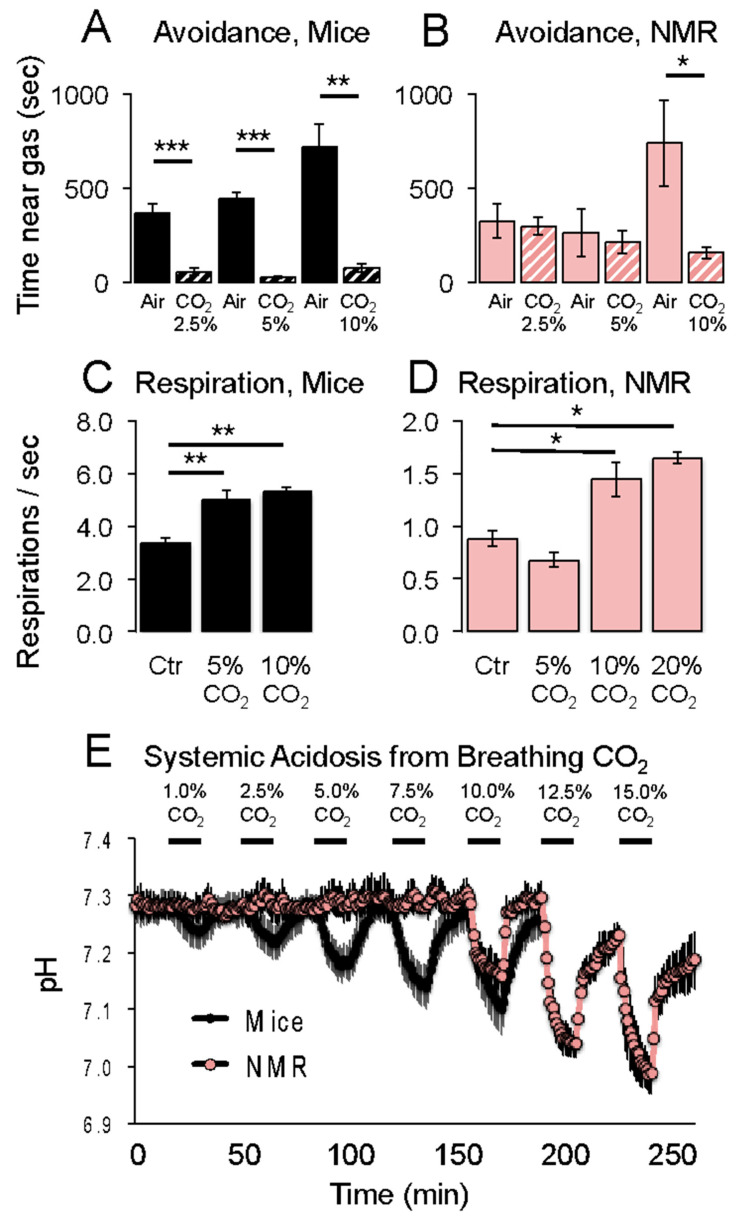
Responses of naked mole-rats and mice to elevated CO_2_ concentrations. Aversion behaviors are shown in (**A**,**B**). Respiration rates are shown in (**C**,**D**). Systemic acidosis is shown in (**E**). * *p* < 0.05, ** *p* < 0.01, *** *p* < 0.001. These graphs are from [19].

Room air contains about 0.03% CO_2_. Breathing higher concentrations of CO_2_ causes acidification of the tissues, with a variety of associated behavioral and physiological responses. For example, humans and laboratory mice find elevated CO_2_ concentrations to be painful to the upper respiratory tract [45,46]. On the other hand, naked mole-rats are less averse to elevated CO_2_ concentrations compared to mice. In an avoidance test, naked mole-rats and mice were placed in a rectangular arena where CO_2_ was infused at one end and room air at the other [19]. In that study, mice avoided all three of the CO_2_ concentrations tested: 2.5%, 5%, and 10% (Figure 8A), whereas naked mole-rats only avoided the highest concentration of 10% CO_2_ (Figure 8B), implying that the naked mole-rats did not find 2.5% or 5% CO_2_ to be aversive.

Elevated CO_2_ concentrations also trigger an increase in respiration rate [46,47]. This can be observed in mice for 5% and 10% CO_2_ (Figure 8C). Naked mole-rats only showed an increase in respiration rate of 10% and greater concentrations of CO_2_ (Figure 8D).

Johansen et al. [48] showed that naked mole-rat blood can buffer acid better than mouse blood. Consistent with this observation, Park et al. [19] showed that naked mole-rats do not show systemic acidification from breathing CO_2_ concentrations below 10%, whereas mice show acidification from breathing even 1% CO_2_ (Figure 8E).

**Figure 9:**
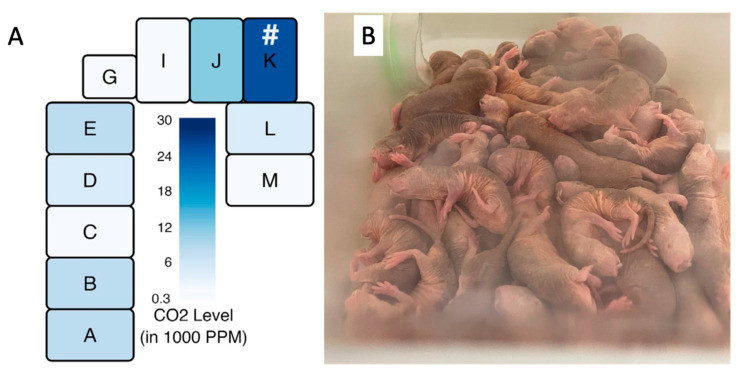
Measured CO_2_ levels of a naked mole-rat cage system as employed by Zions et al. [49] are shown in (**A**). # = location of the nest. The high density of respiring individuals can be seen in a typical nest environment, as seen in a colony housed at the University of Illinois Chicago (**B**), photo by Thomas Park.

Zions et al. demonstrated that captive naked mole-rat habitats have a high degree of anisotropy in carbon dioxide levels that are tightly coupled to the location of the nest chamber [49]. The previously described eusocial nature of this species lends itself to individuals spending a higher fractional time respiring in the nest chamber than the surrounding area. This is reflected in a higher carbon dioxide load in the nest chambers and the surrounding areas. Figure 9A shows a drawing of one of the colony caging systems used in their study. The nest chamber, indicated by the hashtag symbol, was the area showing consistently the highest concentration of CO_2_ in the system and is the chamber in which the naked mole-rats prefer to crowd together, despite the high concentration of CO_2_ as shown in Figure 9B.

Hypercapnia has been demonstrated to suppress neuronal activity [50]. Zions et al. report that the neuronal potassium-chloride cotransporter 2 (KCC2) has a loss-of-function point mutation at position 952 where a highly conserved arginine has been mutated to histidine in the naked mole-rat (R952H) [49]. This mutation results in less chloride being extruded from the neuron, altered chloride homeostasis, and ultimately a reduction in inhibitory currents through GABAergic ion channels (gamma-aminobutyric acid receptors). Zions et al., therefore, propose an elegant hypothesis in which hypercapnia’s ability to suppress neural activity acts to shift the excitation-inhibition balance in naked mole-rats’ neuronal circuits and allows them to conserve energy by reducing the expenditure necessary to drive KCC2 [49]. This hypothesis is supported by their finding of a similar mutation (R952C) in the eusocial relative of the naked mole-rat, the Damaraland mole-rat.

**Figure 10:**
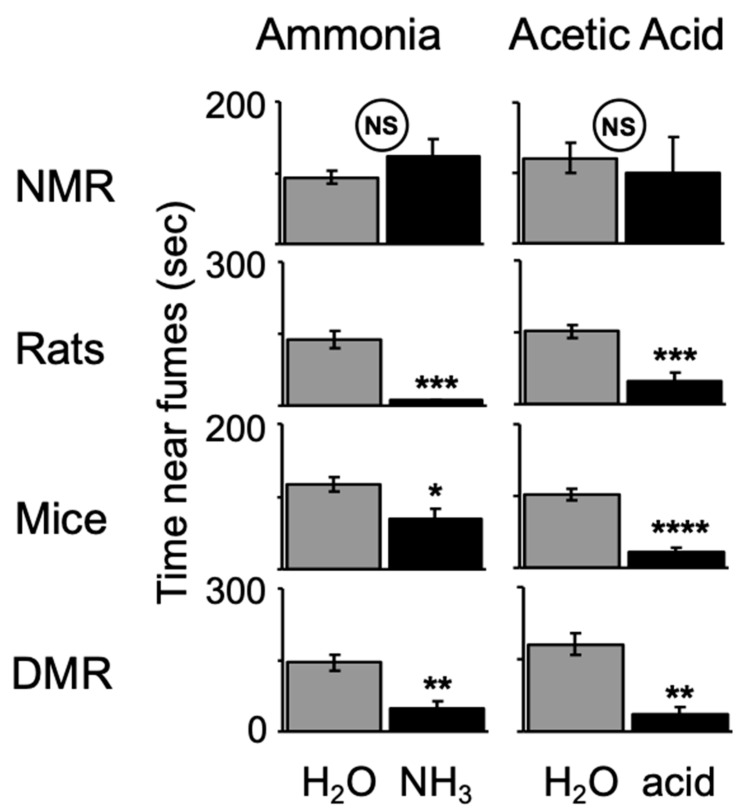
Naked mole-rats (NMR) do not avoid regions containing sponges saturated with noxious irritants creating ammonia or acetic acid fumes, whereas laboratory rats, mice, and Damaraland mole-rats (DMR) significantly avoid these irritants. * *p*< 0.05, ** *p* < 0.01, *** *p* < 0.001, **** *p* < 0.0001. This graph is from [51].

In another study, LaVinka and Park [51] showed that naked mole-rats do not avoid fumes from 10% ammonia (household cleaning ammonia) or 20% acetic acid (Figure 10). In contrast, laboratory rats, mice, and Damaraland mole-rats significantly avoided these irritants.

**Figure 11:**
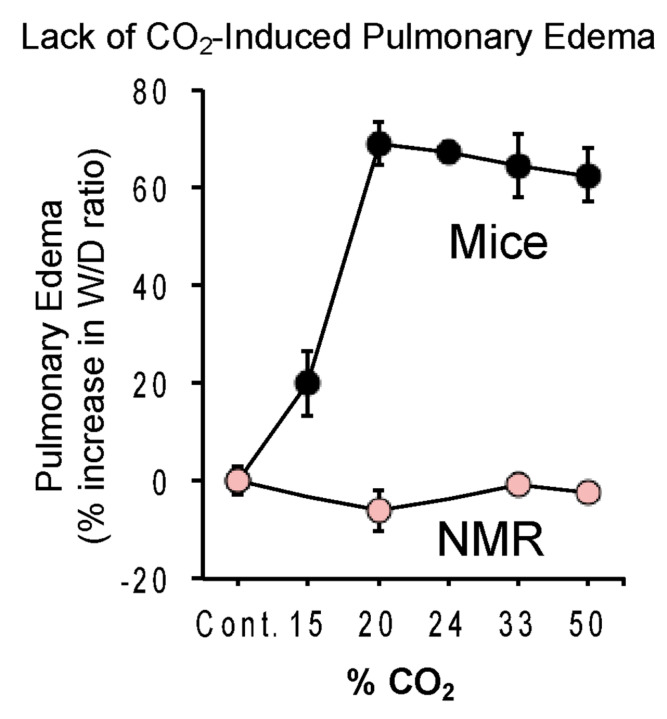
Lack of CO_2_-induced pulmonary edema in naked mole-rats. The curves show the percent change in lung wet—to-dry ratio as a function of the percent CO_2_ in the atmosphere. Animals were exposed to a given atmosphere for 15 min prior to removing and determining both the wet and dry weight of lung tissue. This figure is from [19].

Breathing high concentrations of CO_2_ also induces rapid and life-threatening pulmonary edema. CO_2_ induces changes in the alveolar permeability and reabsorption properties of the lung, and pulmonary edema happens when CO_2_ acidifies the tissues of the lungs, activating acid-sensitive sensory nerves in the tissues that trigger neurogenic inflammation and edema. Park et al. measured pulmonary edema from CO_2_ in naked mole-rats and mice [19]. Naked mole-rats showed no edema even at the highest concentration of CO_2_ tested (50%) (Figure 11). In contrast, mice showed significant edema at 15% and robust edema at 20–50% CO_2_ concentrations.

**Figure 12:**
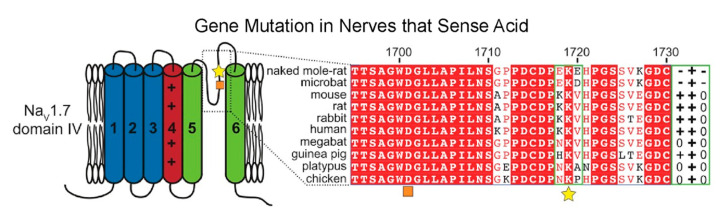
Sequence alignment demonstrating a variant in naked mole-rat Na_V_1.7 that changes the pH sensitivity of the channel. The motif of interest is indicated by a yellow star. The associated charges are indicated at the far right in a green box. The orange square denotes the outer carboxylate ring. This figure is from [23]. This mutation would alter sensitivity to proton accumulation.

A mutation in the voltage-gated sodium channel Na_V_1.7 likely contributes to the lack of CO_2_-induced pulmonary edema in naked mole-rats [23]. Unlike the positive charge of a highly conserved loop motif (the KKV motif in humans) in domain IV observed in most species, this motif in naked mole-rats has a net negative charge (Figure 12). This mutation of residues 1718 and 1720 imparts an altered charge landscape in this region through the placement of two mutated glutamate (E) residues flanking a conserved lysine (the EKE motif). As a result, the sensory nerves that normally respond to acid become significantly less sensitive, blocking the presence of acid-induced neural signaling [23]. The authors suggest that there is a greater level of proton inhibition in naked mole-rat Na_V_1.7 that alters the excitation dynamics of these neurons under acidic conditions. This in turn serves to balance depolarization from TRPV1 (transient receptor potential cation channel subfamily V member 1) and ASIC (acid-sensing ion channel) receptors and inhibit the propagation of this signal via Na_V_1.7. Ultimately, this mutation likely reduces the level of signaling, for under acidic conditions, ASIC receptors will be continually activated by protons. This mutation is likely an evolutionary adaptation to living in an underground hypercapnic niche.

## 4. Insensitivity to Chemical Pain

**Figure 13:**
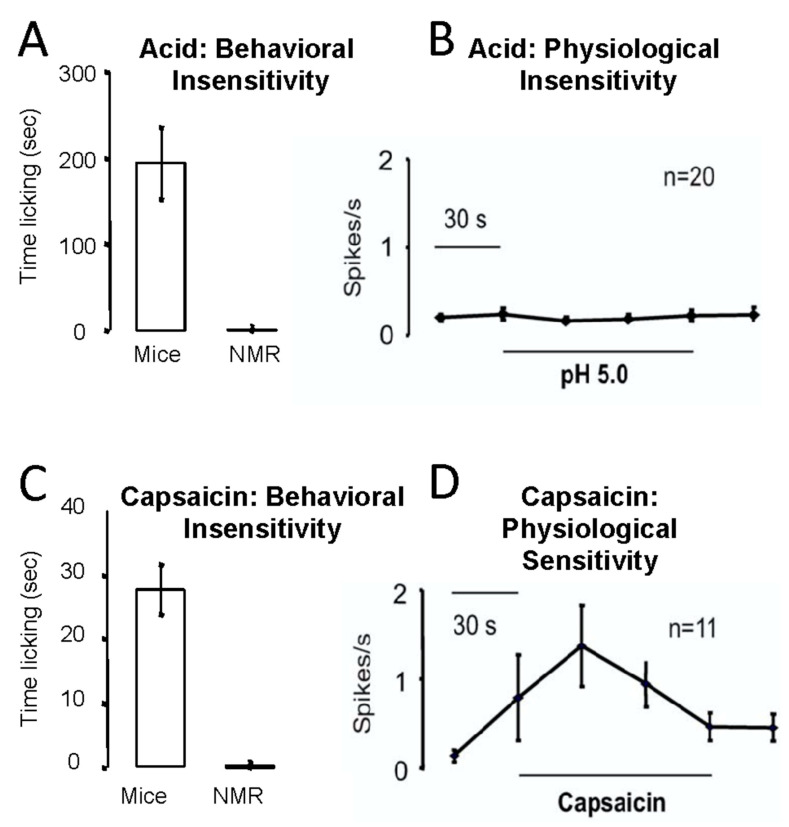
Naked mole-rats are behaviorally (**A**) and physiologically (**B**) insensitive to acid pain. They are behaviorally insensitive to capsaicin pain (**C**), although they showed a physiological response to capsaicin (**D**). The behavioral tests involved injecting an acidic saline or capsaicin solution into the skin of one hind paw. The physiological data were collected from pain-sensing C fibers from the saphenous nerve, using the in vitro skin nerve preparation as described in [52]. These data are from [22].

Consistent with a gene mutation in the nerves that sense acid, naked mole-rats are completely insensitive to acid pain in their skin [22]. Figure 13A shows the behavioral response of mice and naked mole-rats that received an injection of acidic saline in the skin of one hind paw. Mice responded with robust licking of the injection site, whereas naked mole-rats had virtually no response. Consistent with acid insensitivity being associated with a gene mutation, naked mole-rat pain-sensing neurons also showed no response to an acidic bath solution (Figure 13B).

Naked mole-rats were also behaviorally insensitive to the injection of capsaicin, the spicy substance found in “hot” chili peppers (Figure 13C). However, unexpectedly, pain-sensing neurons from naked mole-rats demonstrated robust responses to capsaicin in the bath solution (Figure 13D).

**Figure 14:**
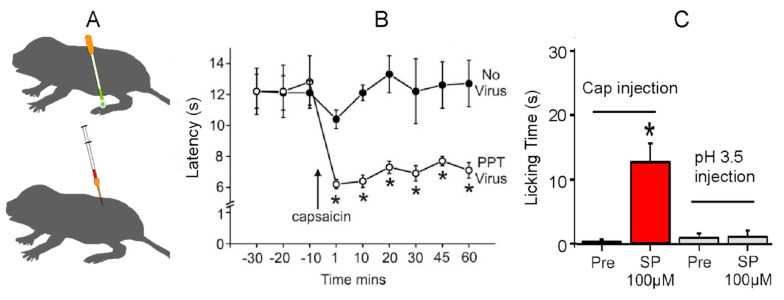
Rescue of capsaicin pain in naked mole-rats from the introduction of exogenous Substance P. (**A**) Application of a transgenic herpes virus expressing the preprotachykinin (PPT/TAC1) gene for Substance P (top) and intrathecal injection of Substance P (bottom). (**B**) This graph is associated with the animals treated with the virus. The graph shows foot withdrawal latency to heat before and after the topical application of capsaicin. The virus-treated foot (PPT Virus) shows sensitization from capsaicin, whereas the “No Virus” foot does not. (**C**) This graph shows the amount of time spent licking the injection site for capsaicin or acid injection before and after an intrathecal application of Substance P. * *p* < 0.05. These data are from [22].

Because the mutation in Na_V_1.7 is specific for sensing acid pain, there must be a different mechanism associated with insensitivity to capsaicin. Park et al. [53] showed that naked mole-rats lack the neuropeptide transmitters Substance P and Calcitonin Gene-Related Peptide from their pain-sensing peripheral nerves. In a subsequent study, Park et al. [22] showed that introducing exogenous Substance P alone was capable of rescuing pain behaviors from capsaicin (Figure 14). The researchers used two techniques to introduce Substance P: the application of a transgenic herpes virus expressing the preprotachykinin (PPT1/TAC1) gene for Substance P (Figure 14A, top) and the intrathecal injection of Substance P directly into the spinal cord (Figure 14A, bottom).

Figure 14B shows pain behavior for the virus-treated foot and the untreated foot (labeled ‘No Virus’ in Figure 14). In this test, the naked mole-rats were lightly anesthetized, and the feet were heated with a calibrated heat source. The first three data points show foot withdrawal latencies from the heat source of about 12 s for both feet. The remaining data points show withdrawal latencies after topical application of capsaicin to both feet. The untreated foot shows withdrawal latencies that are similar to the latencies prior to capsaicin application of about 12 s. However, the virus-treated foot shows robust sensitization to capsaicin in the form of significantly faster withdrawal latencies of about 6 s.

Figure 14C shows the results of introducing Substance P via intrathecal injection. The two bars on the left show the results of testing with capsaicin. In this test, one hind paw was injected with a capsaicin solution prior to Substance P application (labeled Pre in Figure 14). As noted previously, there was virtually no response. However, following the introduction of Substance P, the naked mole-rats spent a significant amount of time licking the injection site, supporting the theory that exogenous Substance P could rescue capsaicin pain. The two bars on the right show the results for acid pain. In this case, the application of Substance P had no effect on acid pain insensitivity, supporting the hypothesis that acid insensitivity was mediated by another mechanism, namely the mutation in Na_V_1.7.

**Figure 15:**
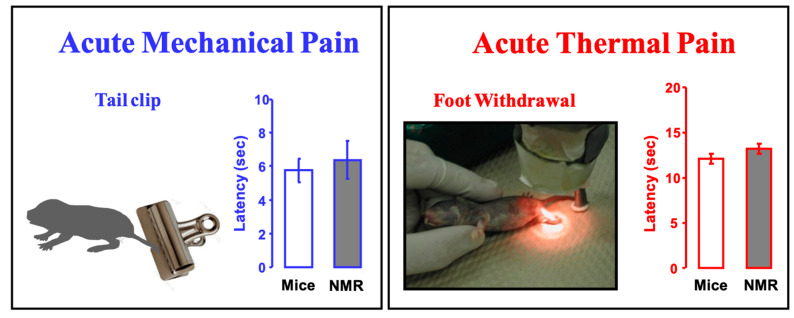
Naked mole-rats show normal (mouse-like) responses to acute mechanical pain (pinch) and acute thermal pain (heat). The bar graphs show latency to respond to a 450 g pinch from a clip at the base of the tail (the clip in the illustration is similar to the one used) or foot withdrawal from a calibrated heat source. These data are from [22].

It is noteworthy that naked mole-rats respond to acute mechanical pain (pinch) and acute thermal pain (heat) in the same way as mice (Figure 15). Both naked mole-rats and mice react to a pinch with a tail clip in about 6 s (Figure 15, left), and both naked mole-rats and mice react to heat pain in about 12 s (Figure 15, right).

**Figure 16:**
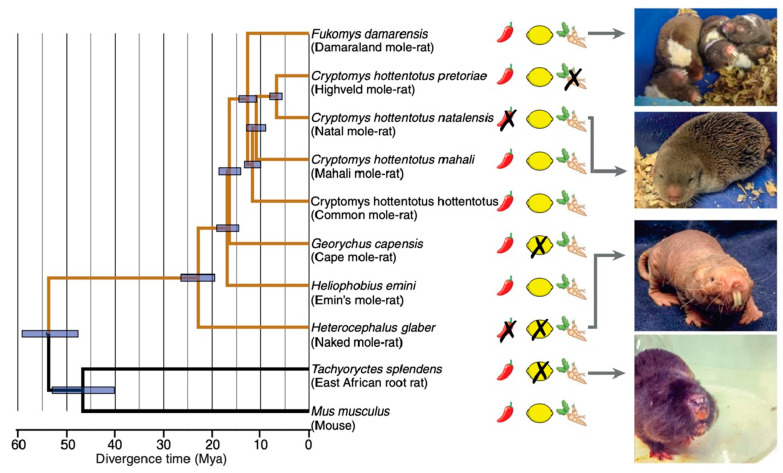
In addition to naked mole-rats, several other African subterranean rodents display insensitivity to capsaicin, acid, or AITC. On the left is a phylogenetic tree for the 10 species tested. Insensitivity is indicated by an “X” over the drawings of a chili pepper, a lemon, or a wasabi root. On the right are photographs of four of the species. This data is from [20].

The naked mole-rat was the first mammal identified to lack acid and capsaicin pain. However, a subsequent study found that some other African subterranean rodents also showed pain insensitivities [20]. Figure 16 shows the results of behavioral tests on eight African mole-rat species, the East African root rat, and the laboratory mouse. Animals were tested with diluted solutions of capsaicin (the chili pepper drawing in Figure 16), acid (the lemon drawing), and allyl isothiocyanate (AITC), the active ingredient in mustard oil and wasabi root (the wasabi root drawing). Pain insensitivity is indicated by an “X” over the drawings. In addition to the naked mole-rat, there was one other species, the Natal mole-rat, that was insensitive to capsaicin, and two other species that were insensitive to acid: the Cape mole-rat and the East African root rat.

An interesting finding was that the Highveld mole-rat, while sensitive to acid burn, was insensitive to AITC, which activates TRPA1 (transient receptor potential cation channel, subfamily A, member 1) receptors [54]. The Highveld mole-rat shares its burrows with the Natal droptail ant, whose normally painful sting is known to activate TRPA1 receptors, likely suggesting a co-evolutionary history. Eigenbrod, et al. [20] showed that, in addition to being insensitive to AITC, the Highveld mole-rat was insensitive to the sting of the Natal droptail ant. Other species tested in the same study showed pain behaviors from the sting. Additional experiments showed that the Highveld mole-rat overexpresses the leak channel NALCN (sodium leak channel, nonselective), which could act to dampen excitation in pain nerves after TRPA1 activation, thus rendering Highveld mole-rats insensitive to the ant’s sting [20].

## 5. Sensory Vibrissae Mediate Orientation to Touch

**Figure 17:**
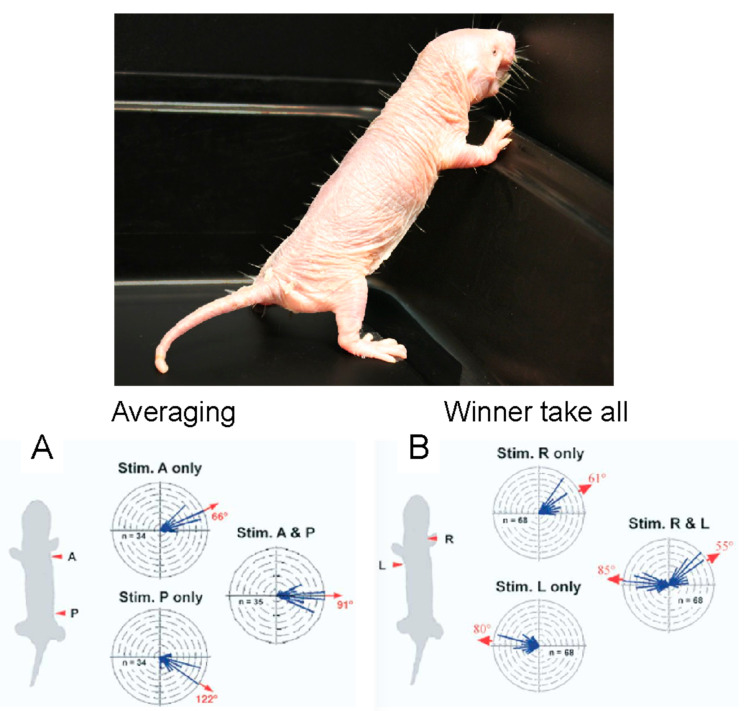
Sensory vibrissae mediate orientation to touch in naked mole-rats. One row of sensory vibrissae can be seen in the photograph of a naked mole-rat against a dark background (photo: Emily Vice). (**A**) Circular histograms show the turn vectors corresponding to orientation responses triggered by deflecting one or two vibrissae on the same side of the animal. (**B**) Circular histograms show the turn vectors corresponding to orientation responses triggered by deflecting one or two vibrissae on opposite sides of the animal. These graphs are from [56].

In addition to pain insensitivities, the somatosensory system of the naked mole-rat has another interesting feature. Although naked mole-rats lack fur, their bodies have 10 rows of sensitive vibrissae that form a sensory array [55]. One row of vibrissae can be seen in the photograph in Figure 17. Crish et al. [55] found that deflecting a single vibrissa triggered an accurate and repeatable orientation of the snout to the position of the deflected vibrissa. Interestingly, simultaneously deflecting two vibrissae on the same side of the body triggered an orientation response to a position that is the average of the two stimulated vibrissae. For example, stimulating a vibrissa at the shoulder and hip triggered an orientation of the snout to a position halfway between the shoulder and the hip (Figure 17A). This result indicates that the naked mole-rat nervous system is capable of making a computation about touch location. However, this is not the case for simultaneously deflecting two vibrissae on opposite sides of the body, which triggers an orientation to either one or the other deflected vibrissa in a winner-take-all strategy (Figure 17B).

## 6. Conclusions

Naked mole-rats have evolved under extraordinary environmental challenges, resulting in the evolution of a suite of exceptional adaptations to deal with those challenges. The current review article highlights some of those adaptations related to the naked mole-rat’s tolerance to hypoxia, hypercapnia, and pain, including some of the underlying mechanisms. Although, there is still much to be learned about naked mole-rat adaptations to the atmospheric conditions under which they have evolved, elucidating the protective mechanisms they employ may highlight new directions for the treatment of human pathological conditions such as inflammation and concomitant pain, as well as ischemia/reperfusion injury as occurs in cardiac infarctions and stroke.
